# To: Heliox in the treatment of status asthmaticus: case reports

**DOI:** 10.5935/0103-507X.20160059

**Published:** 2016

**Authors:** Cayetano Díaz Chantar, João Pedro Abreu Cravo, Antonio M. Esquinas

**Affiliations:** Servicio de Neumología, Hospital Universitario Morales Meseguer - Murcia, España; Servicio Pneumología A, Centro Hospitalar e Universitário de Coimbra - Portugal; Unidad de Cuidados Intensivos, Hospital Universitario Morales Meseguer - Murcia, España

**To the Editor**

Bronchodilators and corticosteroids are used in clinical practice^(1)^ to increase airway caliber and decrease resistance, wherein heliotherapy (heliox) may play key role. Heliox is insoluble in human tissues and has no bronchodilator or anti-inflammatory effects. However, its low density (one tenth of air density) enables a decrease in airflow resistance, regardless of any anatomical change; thus, the flow is less turbulent and the ventilatory process is more efficient.

Carvalho et al.^(2)^ recently published a study in your journal (Revista Brasileira de Terapia Intensiva) entitled "Heliox in the treatment of status asthmaticus: case reports". The authors reported 2 clinical cases and reviewed some clinical data published in the literature that suggest that heliox^(3)^ may play a key role in the treatment of these patients and perhaps other obstructive airway diseases, despite being included in clinical practice guidelines.

We found the article very interesting, so we would like to make some comments:

First, static (compliance) and dynamic (airflow resistance) properties govern respiratory mechanics. Fluid mechanics has shown that the flow through a vessel may be laminar, turbulent or mixed and is determined by the Reynolds number, which is proportional to the product of the airway diameter, the flow rate and the gas density divided by the viscosity. Therefore, heliox alone decreases airflow resistance because of its low density; this effect is further enhanced by the gradual decrease in diameter along the airway (decreasing the Reynolds number to a value < 2000), leading to improved ventilation and decreased respiratory effort.

Second, the authors emphasize the importance of using heliox (80/20) because other mixtures with a lower proportion of helium would increase the mixture density, thereby causing a more turbulent flow. The authors recommend using a Maquet Servo-I ventilator to obtain this proportion because its volume quantification is more accurate. However, some questions remain. How reliable are the volume values? How does heliox affect the dosing of aerosols? Lastly, would it be advisable/beneficial to use a humidifier system?

Lastly, we would like to discuss two issues regarding the selected image. The inspiratory (I) portion seems very long, almost 3 times the exhalation (E) portion, which is not the most suitable I:E ratio for an asthma attack, and the respiratory rate and volumes are somewhat low. The positive end-expiratory pressure (PEEP) of zero^(4,5)^ would be nonphysiological in patients with obstructive airway diseases. It would be more appropriate to recruit more alveoli rather than fewer alevoli, which would lead to increases in the shunt effect and respiratory effort.

Cayetano Díaz Chantar

Servicio de Neumología, Hospital Universitario Morales Meseguer - Murcia, España

João Pedro Abreu Cravo

Servicio Pneumología A, Centro Hospitalar e Universitário de Coimbra - Portugal

Antonio M. Esquinas

Unidad de Cuidados Intensivos, Hospital Universitario Morales Meseguer - Murcia, España
